# CCL17‐expressing dendritic cells in the intestine are preferentially infected by *Salmonella* but CCL17 plays a redundant role in systemic dissemination

**DOI:** 10.1002/iid3.445

**Published:** 2021-05-04

**Authors:** Anna B. Erazo, Nancy Wang, Lena Standke, Adrian D. Semeniuk, Lorenz Fülle, Sevgi C. Cengiz, Manja Thiem, Heike Weighardt, Richard A. Strugnell, Irmgard Förster

**Affiliations:** ^1^ Immunology and Environment Life and Medical Sciences (LIMES) Institute, University of Bonn Bonn Germany; ^2^ Department of Microbiology and Immunology The University of Melbourne at the Peter Doherty Institute for Infection and Immunity Melbourne Victoria Australia; ^3^ Department for Innate Immunity and Metaflammation Institute of Innate Immunity, University Hospital Bonn, Medical Faculty Bonn Germany

**Keywords:** CCL17, dendritic cells, lymph node, Peyers's patch, *Salmonella*, typhoid fever

## Abstract

**Introduction:**

*Salmonella* spp. are a recognized and global cause of serious health issues from gastroenteritis to invasive disease. The mouse model of human typhoid fever, which uses *Salmonella enterica* serovar Typhimurium (STM) in susceptible mouse strains, has revealed that the bacteria gain access to extraintestinal tissues from the gastrointestinal tract to cause severe systemic disease. Previous analysis of the immune responses against *Salmonella* spp. revealed the crucial role played by dendritic cells (DCs) in carrying STM from the intestinal mucosa to the mesenteric lymph nodes (mLNs), a key site for antigen presentation and T cell activation. In this study, we investigated the influence of chemokine CCL17 on the dissemination of STM.

**Methods:**

WT, CCL17/EGFP reporter, or CCL17‐deficient mice were infected orally with STM (SL1344) or mCherry‐expressing STM for 1–3 days. Colocalization of STM with CCL17‐expressing DCs in Peyer's patches (PP) and mLN was analyzed by fluorescence microscopy. In addition, DCs and myeloid cell populations from naïve and *Salmonella*‐infected mice were analyzed by flow cytometry. Bacterial load was determined in PP, mLN, spleen, and liver 1 and 3 days after infection.

**Results:**

Histological analysis revealed that CCL17‐expressing cells are located in close proximity to STM in the dome area of PP. We show that, in mLN, STM were preferentially located within CCL17^+^ rather than CCL17^−^ DCs, besides other mononuclear phagocytes, and identified the CD103^+^ CD11b^−^ DC subset as the main STM‐carrying DC population in the intestine. STM infection triggered upregulation of CCL17 expression in specific intestinal DC subsets in a tissue‐specific manner. The dissemination of STM from the gut to the mLN, however, was only moderately influenced by the presence of CCL17.

**Conclusion:**

CCL17‐expressing DCs were preferentially infected by *Salmonella* in the intestine in comparison to other DC. Nevertheless, the production of CCL17 was not essential for the early dissemination of *Salmonella* from the gut to systemic organs.

## Graphical abstract

Oral infection with *Salmonella* enhances the frequency of dendritic cells expressing the pro‐inflammatory chemokine CCL17, promoting dendritic cell–T cell interaction. In Peyer's patches, CCL17‐expressing cells colocalize with *Salmonella* and *Salmonella*‐containing cells in mesenteric lymph nodes are preferentially CCL17‐positive. While CCL17‐expressing cells participate in the early dissemination of *Salmonella*, CCL17 itself appears to play a redundant role in this process.

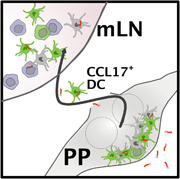

## 1 INTRODUCTION

*Salmonella enterica* infect a wide range of animals, from mammals, birds to reptiles and have been a recognized cause of infectious diseases in humans for centuries. *Salmonella* is generally acquired through the ingestion of contaminated food or water. The bacterium survives passage through the stomach and replicates in the small intestine.^1^ In humans, the diseases caused by *S. enterica* range from asymptomatic carriage to a potentially fatal systemic infection. *S*. *enterica* serovars Typhi and Paratyphi cause typhoid and paratyphoid fever, respectively, which present as febrile, systemic infections,^2^ with over 20 million new cases and 120,000 deaths annually. In contrast, non‐typhoidal *Salmonella* serovars such as *S*. *enterica* serovar Typhimurium (STM) and serovar Enteritidis (STE) are a frequent cause of gut‐associated gastroenteritis in developed countries.^2^ More recently, a subset of STM and STE strains have been linked to invasive non‐typhoidal Salmonellosis (iNTS) in sub‐Saharan Africa with 20%–25% mortality.^3^, ^4^, ^5^ An experimental model of iNTS can be reproduced by oral infection of susceptible mouse strains with STM.^6^

Following oral ingestion, STM crosses the protective epithelial barrier through phagocytic M cells in the follicle‐associated epithelium (FAE) overlying the Peyer's patches (PP). In addition, STM can induce its own uptake by non‐phagocytic enterocytes through its virulence‐associated type 3 secretion system (T3SS) encoded by *Salmonella* Pathogenicity Island 1 (SPI‐1). The third route of invasion is via trans‐epithelial sampling by mononuclear phagocytes that reside immediately on the basal side of the gut epithelial linings.^7^, ^8^, ^9^, ^10^, ^11^

Independent of the specific mechanism(s) by which STM enters the host gut tissue, the processes that enable STM to spread from the intestine to the draining lymph nodes (LN), and on to the circulation, are a key focus in understanding the systemic disease caused by STM. In situ, STM is found inside a range of phagocytic cells including dendritic cells (DCs),^12^, ^13^, ^14^ neutrophils,^14^, ^15^, ^16^ inflammatory monocytes,^15^ and macrophages (Mφ).^14^, ^15^, ^17^ Careful analysis of infected tissues indicates that the great majority of viable bacteria in tissues are found within Mφ.^15^, ^18^, ^19^ It is also clear that phagocytes affect STM in different ways. Neutrophils and inflammatory monocytes exhibit potent antibacterial activity.^15^ In contrast, STM‐containing Mφ has been associated with a reduced ability to kill intracellular bacteria.^15^, ^17^ In STM‐infected DCs, SteD‐dependent downregulation of surface MHCII results in suppression of antigen presentation and T cell activation.^20^ Moreover, STM has been observed in some B cells within the mesenteric LN (mLN), but only DCs have been shown to carry viable STM from the intestine through the lymphatics to the mLN.^14^

Cell migration and antigen presentation are critical steps in controlling systemic dissemination of a pathogen and the induction of adaptive immunity, especially where the pathogen typically resides intracellularly. The chemokine CCL17, a ligand of CCR4, was originally identified in the thymus^21^ and later described in a subset of conventional DC (cDC) in different barrier organs such as the gut and the skin and their draining LN.^22^ In vitro and in vivo studies suggest that the major functions of CCL17 include the induction of T cell chemotaxis via the receptor CCR4,^23^, ^24^ the initiation of DC–T cell interactions^25^ and the support of DC migration.^26^ CCL17 promotes cutaneous DC migration from the skin by enhancing responsiveness to the CCR7 (CCL19/21) and CXCR4 (CXCL12) ligands.^26^ In the immune system, CCL17 is classified as a dual‐function chemokine, exhibiting both homeostatic and inflammatory functions.^27^ In mouse models, CCL17 is involved in the induction or enhancement of a broad spectrum of inflammatory and allergic diseases, including atopic dermatitis, colitis, and arthritis.^22^, ^23^, ^24^, ^26^, ^28^, ^29^ In contrast to inflammatory and allergic diseases, the role of CCL17 in infectious diseases is less well understood.

In this study, we aimed to investigate the role of CCL17 during the early phase of infection, including bacterial spreading from the intestine to the draining LN. We demonstrate that the frequency of CCL17^+^ cells increases after STM infection and that STM^+^ DCs contain a higher proportion of CCL17^+^ cells than noninfected DCs. Nevertheless, the early dissemination of STM to systemic organs was only marginally affected by the presence of CCL17.

## 2 MATERIALS AND METHODS

### 2.1 Mice

Mice were housed under specific pathogen‐free (SPF) conditions in the Genetic Resources Center (GRC) of the Life & Medical Sciences (LIMES) Institute, University of Bonn, Germany, or in the Biological Research Facility of the Department of Microbiology and Immunology, The University of Melbourne, at Peter Doherty Institute for Infection and Immunity, Australia. All animal experiments were performed with age‐ and sex‐matched mice using male or female 8–14 weeks old wild‐type (wt) (C57BL/6J‐RCCHsd), CCL17^E/+,^ or CCL17^E/E^ mice. CCL17^E/+^ and CCL17^E/E^ mice were generated as previously described^22^ and backcrossed onto the C57BL/6J‐RCCHsd background. Both CCL17^E/+^ and CCL17^E/E^ mice express the enhanced green fluorescent protein (EGFP) under control of the endogenous *Ccl17* promoter from either one or both alleles. All mice were bred in‐house. Animal experiments performed in Bonn were approved by the government of North Rhine‐Westphalia, Germany (Az 84‐02.04.2013.A084, Az 81‐02.04.2018.A094). Experiments performed in Melbourne were approved by the University of Melbourne Animal Ethics Committee (AEC) (project 1613898), and complied with the National Health and Medical Research Council (NHMRC) Australian Code of Practice for the Care and Use of Animals for Scientific Purposes (8th edition, 2013).

### 2.2 Bacterial infection

All STM strains used were derived from wt STM SL1344. For infection, a single bacterial colony was grown in Luria–Bertani (LB) broth shaking at 160 rpm and at 37°C overnight. The following day, the overnight culture was subcultured in fresh LB broth at 1:100 and grown for 3 h shaking at 160 rpm and at 37°C. Bacteria were harvested by centrifugation (3700 rpm for 10 min) followed by two washing steps with sterile phosphate‐buffered saline (PBS). The concentration of bacteria was adjusted to 1–2.5 × 10^9^ CFU wt STM or mCherry STM^30^ in a volume of 200 μl of LB broth and 10% sodium bicarbonate mixed at 1:1 ratio. Mice were infected via oral gavage.

### 2.3 Microscopy

For immunohistochemistry of mLN and PP, tissue samples were fixed overnight in 4% paraformaldehyde (PFA), dehydrated for 3 h in a 30% sucrose solution, embedded in Tissue‐Tek™ Cryomold™ with tissue freezing medium and stored at −20°C. Frozen samples were cut into 10‐μm thick sections using a Leica CM3050 S cryostat, transferred to specimen slides, fixed in acetone for 5 min, washed in PBS, air‐dried, and finally stored at −80°C. Samples were thawed immediately before staining and rehydrated in PBS for 5 min. Slides with samples were placed in a humidified chamber for blocking and staining. Tissue sections were covered with histo block (PBS, 1% BSA, 2% donkey serum, 2% goat serum) and incubated at RT for 2 h. Then samples were subjected to staining with primary antibodies (polyclonal rabbit anti‐RFP [Rockland], polyclonal rat anti‐EGFP [BioLegend]) in PBS with 1% fetal calf serum (FCS) overnight at 4°C. Sections were washed with PBS and then covered with secondary antibodies (polyclonal anti‐rabbit IgG and anti‐rat IgG [Thermo Fisher Scientific]) in PBS with 1% FCS for 2 h at RT. Subsequently, samples were washed with PBS, stained with DAPI (1:1000) for 10 min at RT, and washed again. Finally, they were covered with Mowiol and coverslips, and stored at 4°C. Images of stained samples were acquired using the Keyence BX9000 or the LSM 780 ZEISS for confocal images and analyzed with ImageJ (Rasband, W.S., ImageJ, U.S. National Institutes of Health).

### 2.4 Cell isolation

The mLN and small intestines (SIs) were collected in ice‐cold HBSS with 10% FCS. SI was flushed with ice‐cold PBS and PP was extracted. Remaining fat tissue was removed from mLN. Organs were stored in ice‐cold HBSS with 10% FCS. Organs were pretreated with 100 μg/ml gentamicin for 30 min at RT and then washed with PBS. Next, organs were digested in digestion buffer (RPMI with 5% FCS, 100 U/ml Liberase™ [Merck], and 5 U/ml DNase [Merck]) for 20 min shaking at 37°C, then pushed through a 70 μm cell strainer to generate single‐cell suspension. Finally, single cells were filtered, washed with FACS buffer, and resuspended in FACS buffer for further processing.

### 2.5 Flow cytometry

Before cell surface staining, samples were incubated with Fc‐block™ (BD Biosciences). Staining was performed in PBS with 2% FCS and 5 mM EDTA for 45 min on ice. A combination of fluorochrome‐conjugated monoclonal antibodies was used, specific to mouse CD19 (PE‐Cy7 – clone eBio1D3), CD3 (AF700 – clone 17A2) or TCRβ (PE‐Cy7 – clone H57‐597), CD11b (BV711 – clone M1/70), CD11c (BV786 – clone HL3), CD45 (BV605 – clone 30‐F11), CD103 (BV421 – clone M290), CD64 (AF647 – clone X54‐5/7.1), Ly6C (BV605 – clone AL21), and MHCII (PerCP‐Cy5.5 – clone M5/114.15.2) and were purchased either from BD Biosciences, BioLegend, or eBioscience. To determine cell viability, fixable viability dye eFluor 780 (eBioscience) was added to samples. To detect intracellular STM, viable cells were stained for the surface markers, fixed, and permeabilized using the eBioscience™ Foxp3/Transcription Factor Staining Buffer Set (eBioscience) according to the manufacturer's protocol and incubated with a PE‐labeled anti‐*Salmonella*‐LPS monoclonal antibody (clone 1E6; Abcam). In fixed cells, EGFP was counterstained with an AF488 labeled polyclonal anti‐GFP antibody (Abcam). Data was collected using a BD™ LSR II flow cytometer or a BD LSRFortessa™ cell analyzer. Data analysis was done using the FlowJo™ software.

### 2.6 Bacterial recovery

The numbers of inoculated bacteria and bacterial load in tissues were determined by viable plate count. Livers and spleens were dissected and homogenized using a stomacher (Seward). For PP and mLN single‐cell preparations as described above (cell isolation) were used. Bacteria present in tissues were quantified by plating serial dilutions on LB agar. LB agar was supplemented with streptomycin 50 μg/ml for wt SL1344, and chloramphenicol 100 μg/ml for the experiments using mCherry STM.

### 2.7 Statistical analysis

Data were analyzed with GraphPad Prism 6 (GraphPad Software) using unpaired *t*‐test for comparison between two groups, or one‐way ANOVA with Tukey's posttests and two‐way ANOVA with Bonferroni's posttests for multiple comparisons. Statistical significance was denoted as not significant (ns) *p* > .05, *p < .05, **p < .01, ***p < .001, and ****p < 0.0001 as indicated in the figure legends.

## 3 RESULTS

### 3.1 CCL17‐expressing cells are located in close proximity to *Salmonella* in the dome area of PP

**Figure 1:**
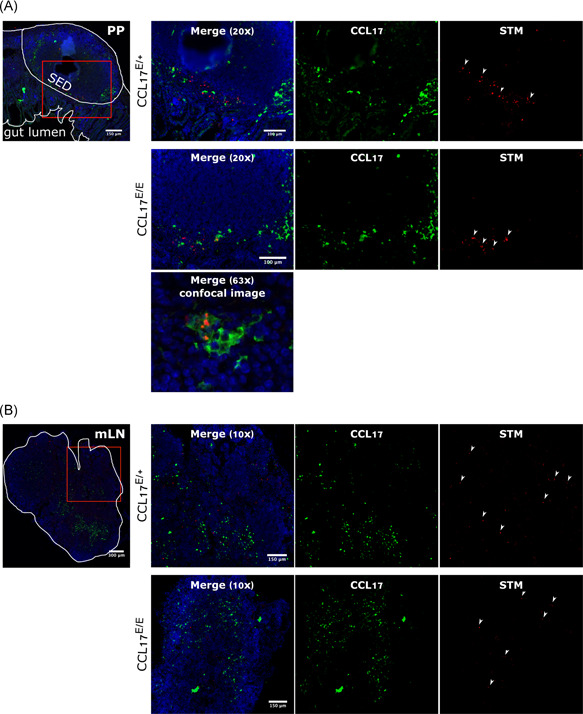
CCL17‐expressing cells localize together with *Salmonella* Typhimurium in the dome area of Peyer's patches. CCL17^E/+^ and CCL17^E/E^ mice were orally gavaged with 2.5 × 10^9^ STM expressing mCherry. PP and mLN were isolated 3 days postinfection, fixed, and subjected to immunofluorescent staining. Images of vertical sections through the PP (A) and through mLN (B) were analyzed via fluorescent microscopy. Shown are CCL17^+^/EGFP^+^ cells (green), STM (red), and nuclei (blue) in pseudo‐color. First left panel depicts a representative structure of the analyzed organ; red box indicates analyzed area of organ. Pseudo‐color merged images are depicted in the panels on the left. Scale bars are included in the merged image. White arrows indicate STM (*n* = 9), three independent experiments were performed, representative images are shown. 20× images were taken on a Keyence BX9000 digital microscope. The 63× image shown in (A) is from a different area of the same section (CCL17^E/E^) and was taken on a confocal microscope

During STM infection, microfold (M) cells and PP are the main entry points for bacteria from the GI tract,^1^, ^31^ where they are taken up by DC and other cells.^10^, ^32^ PP are also a site where CCL17^+^ cells are known to accumulate.^22^ These observations suggest potential interactions between CCL17^+^ cells and STM in the PP and in mLN. In this study, CCL17^E/+^ reporter mice and CCL17^E/E^ (i.e., CCL17 knockout) mice were orally gavaged with mCherry‐expressing STM. Due to a gene dosage effect, CCL17^E/+^ mice which carry one target‐disrupted and one wt *Ccl17* allele produce approximately half the amount of CCL17 as observed in wt mice, whereas CCL17^E/E^ mice express the EGFP reporter from both targeted alleles and are deficient for CCL17. Thus, CCL17‐expressing cells were identified by green fluorescence and bacteria by red fluorescence. PP and mLN were analyzed via immunofluorescent microscopy. Histological analysis of PP sections from CCL17^E/+^ and CCL17^E/E^ mice showed STM and EGFP/CCL17‐expressing cells located in close proximity to each other in the subepithelial dome region (SED) of PP (Figure 1A). In some cases, STM was found inside of EGFP^+^ cells (Figure 1A). In contrast to observations made in the PP, the distribution of bacteria in the mLN of CCL17^E/+^ and CCL17^E/E^ mice was not restricted to the areas where EGFP^+^/CCL17^+^ cells were localized; STM expressing mCherry were detected throughout the mLN (Figure 1B). These findings demonstrate that CCL17‐expressing cells are located in the vicinity of STM in the SED area of PP and in some cases STM can also be detected within CCL17^+^ cells. The absence of CCL17, however, did not interfere with the localization of EGFP^+^ cells, or STM, in PP or mLN.

### 3.2 CCL17‐expressing DC harbor *Salmonella* Typhimurium in mLN

**Figure 2:**
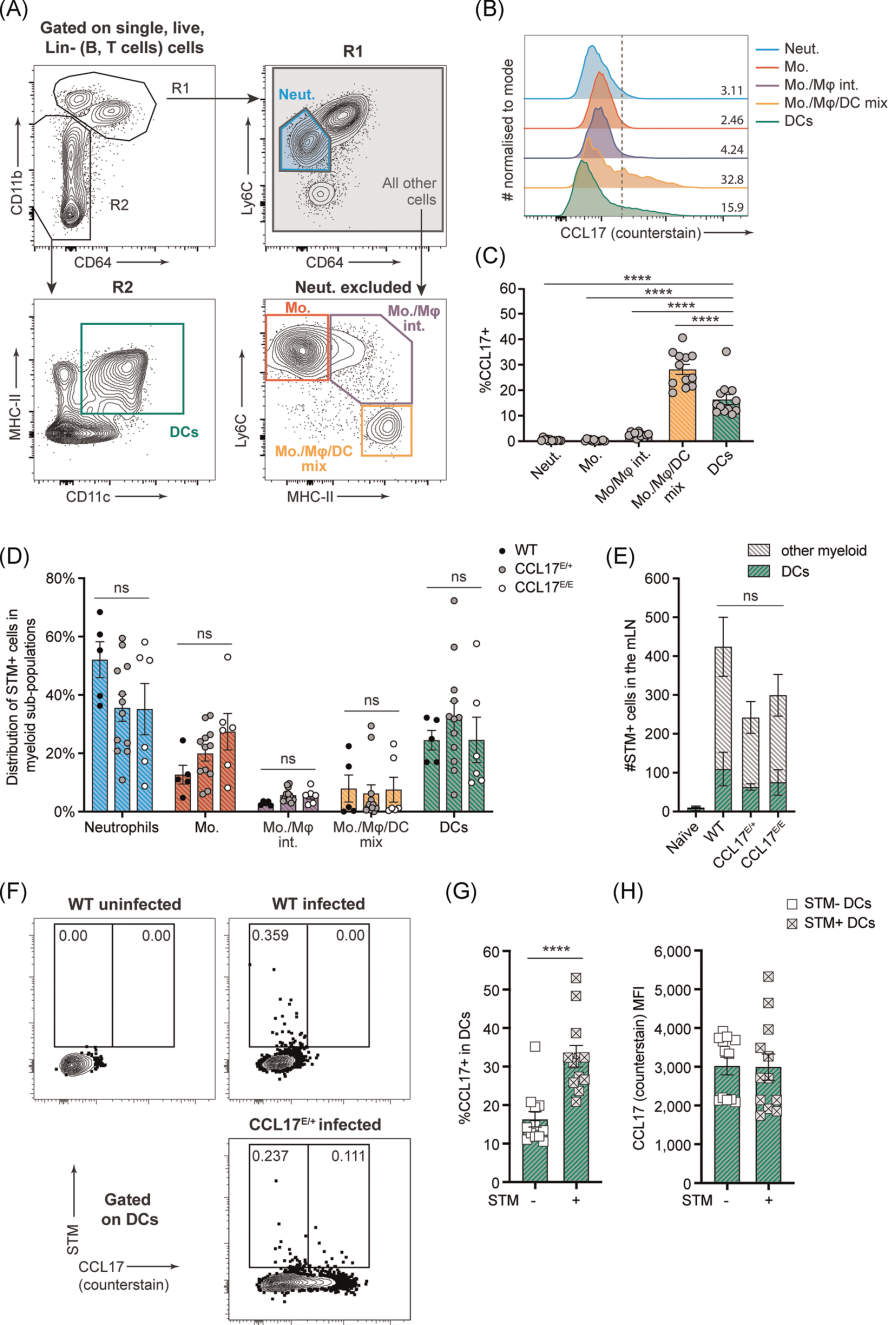
CCL17‐expressing DC harbor *Salmonella* Typhimurium in the mLN. Wt, CCL17^E/+^, and CCL17^E/E^ mice were infected with 1 × 10^9^ STM via oral gavage. mLN were isolated from 36‐h‐infected mice and control naïve mice; mononuclear phagocytes were analyzed via flow cytometry. Shown is the representative gating strategy on a CCL17^E/+^ mouse at 36 h postinfection (A). Representative FACS plots demonstrating CCL17/EGFP expression as analyzed by intracellular counterstain against EGFP are shown for DC, neutrophils (Neut.), monocytes (Mo.), monocyte/macrophage intermediates (Mo./Mφ int), and monocyte/macrophages/DC (Mo./Mφ/DC) mix populations (B) and the frequency of CCL17^+^ cells in these cell populations was compared using one‐way ANOVA with Tukey's posttest. The gate used for determination of CCL17^+^ cells is indicated by a vertical line (C). The distribution of intracellular STM within individual cell populations was calculated as percentage of total STM^+^ cells (i.e., sum of all myeloid STM^+^ cells), and statistical significance was analyzed using one‐way ANOVA with Tukey's posttest (D). The number of STM^+^ cells was quantified within the DC subset (green) and the remainder of the myeloid compartment (gray). Statistical significance was analyzed using two‐way ANOVA with Bonferroni's posttest (E). Representative FACS plots show that approximately one‐third of STM^+^ DCs co‐express CCL17 (F). Within STM^+^ and STM^−^ DCs, the percentage of CCL17‐expressing cells (G) and the MFI of CCL17 expression in CCL17^+^ cells (H) were analyzed, and an unpaired t‐test was used to analyze statistical significance. Error bars indicate mean ± *SEM*. To minimize statistical bias, samples with fewer than 20 STM^+^ DCs were excluded from all analyses; data shown include WT *n* = 5, CCL17^E/+^ *n* = 12, CCL17^E/E^ *n* = 6, pooled from five independent experiments

Since STM and CCL17‐expressing cells reside in very close proximity to each other in the SED area of PP, it was of interest to investigate whether CCL17^+^ DCs might be specifically targeted by STM. Several studies have investigated the cell populations responsible for the transport and therefore spreading of *Salmonella* from the gut to the mLN. DCs have been identified to be ´highjacked ´ by STM for intracellular transportation from the intestine to the mLN.^12^, ^14^, ^33^ In addition to DCs, Mφ, and monocytes can also ingest STM after in vivo infection,^14^, ^34^ but only DCs have been proven to carry viable bacteria from the gut to the draining lymph nodes.^14^ To assess whether CCL17^+^ cells are preferentially infected by STM and whether the frequency of STM^+^ cells in mLN is influenced by CCL17, Wt, CCL17^E/+^ reporter, and CCL17‐deficient CCL17^E/E^ mice were orally gavaged with STM SL1344. mLN were isolated 36 h postinfection and the mononuclear phagocyte (MNP) compartment was initially analyzed for CCL17/EGFP expression. For these analyses, we isolated the entire string of mLN located adjacent to the proximal colon in the mesentery, some of which drain the SI and some the proximal colon.^35^ As shown in Figure 2A, viable, single, Lin^−^ (T and B cells excluded) cells were first separated based on their level of CD11b, a common marker found on myeloid cells including neutrophils, monocytes, and Mφ, as well as a subset of DCs. Within the CD11b^hi^ (R1) gate, Ly6C^int^CD64^neg^ cells were identified as neutrophils (Figure S1), and the remaining cells were further characterized based on Ly6C and MHCII expression using “waterfall” gates, as monocytes (Ly6C^hi^MHCII^neg^), monocyte/Mφ intermediates (Ly6C^hi^MHCII^hi^) and monocyte/Mφ/DC mixed population (Ly6C^neg^MHCII^hi^). Cells that were CD64^neg^CD11b^int/neg^ (R2) were further analyzed for CD11c and MHCII expression, those that were CD64^neg^CD11c^+^MHCII^+^ were identified as DCs (Figure 2A). Based on this gating strategy, DCs and monocytes were among the most abundant myeloid cells in the mLN at 36 h postinfection with STM (Figure S2). Notable CCL17 expression was only observed in DCs and the Ly6C^neg^MHCII^hi^ mixed population. The latter population contained more than 70% CD11c^+^ cells, presumably Mφ or DCs (Figure 2B,C and data not shown), but represented only a very small fraction of cells in the mLN (Figure S2). This cell population appears to express low levels of CD64 and high levels of MHCII and, therefore, may also contain the recently described inflammatory cDC2, which are CD64^lo^CD26^+^MHCII^+^.^36^ As we did not include CD26 in the staining panel, however, we cannot precisely assign the identity of these cells. We evaluated intracellular bacteria using an antibody specific to STM‐LPS, since the antibody proved to be more sensitive in flow cytometry than the mCherry‐expressing STM used for histological analysis. STM^+^ cells were detected within all myeloid cell subsets analyzed, where the most abundant STM^+^ cells were neutrophils, monocytes, and DCs (Figure 2D). As we excluded B and T cells in the lineage gate, we cannot draw a conclusion about the proportion of STM^+^ cells in lymphocytes. The STM^+^ cell count was not significantly affected by the genetic background of mice (Figure 2D,E), indicating that CCL17‐deficiency did not alter the frequency of STM^+^ cells in the mLN. Furthermore, the correlation between CCL17/EGFP expression and presence of STM in DCs was assessed. Approximately one‐third of STM^+^ DC expressed CCL17, whereas the proportion of CCL17^+^ cells was significantly lower in STM^‐^ DCs (Figure 2F,G). The overall level of CCL17 expression was similar in DCs containing STM compared to uninfected DC (Figure 2H). Together our data show that DC harbored STM within the mLN consistent with previous studies,^12^, ^14^, ^33^ including CCL17^+^ DCs and that a higher proportion of STM‐containing DCs was CCL17^+^ compared to uninfected DCs.

### 3.3 *Salmonella* Typhimurium‐infected cells are more frequently found in the CD103^+^ CD11b^−^ DC subset

**Figure 3:**
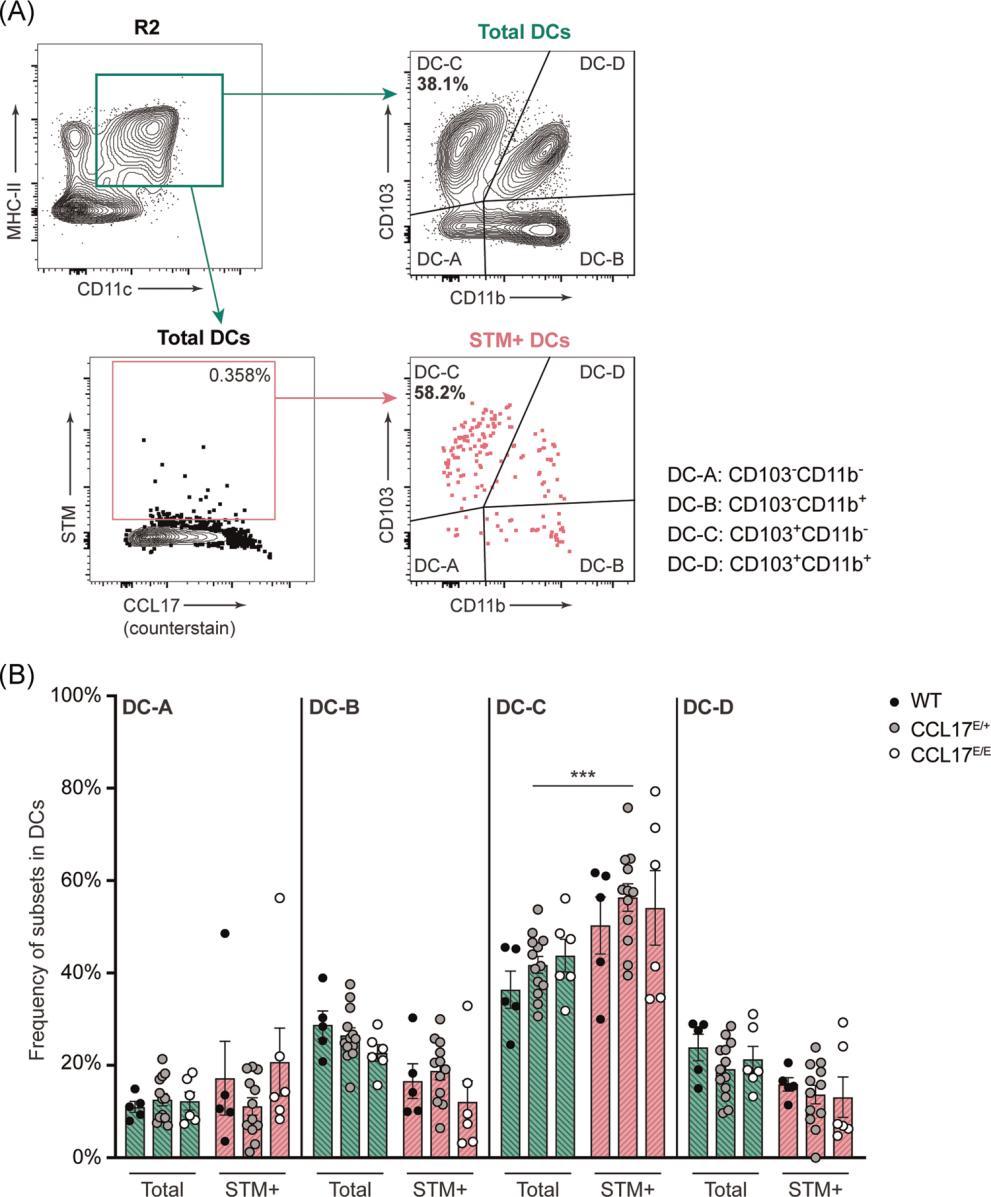
*Salmonella* Typhimurium targets CD103^+^ CD11b^−^ DC. Wt, CCL17^E/+^, and CCL17^E/E^ mice were orally gavaged with 1 × 10^9^ STM. mLN were isolated from 36‐h‐infected mice and single‐cell suspensions were stained for DC surface markers and intracellular *Salmonella*. DC were identified as MHCII^+^ CD64^−^ CD11c^+^ cells. They were further subdivided into four subpopulations depending on their expression of CD103 and CD11b, and STM^+^ DC subpopulations were evaluated. Representative FACS plots show CD103 and CD11b expression in total DCs and in STM^+^ DCs (A). The frequency of DC‐A (CD103^− ^CD11b^−^), DC‐B (CD103^−^ CD11b^+^), DC‐C (CD103^+^ CD11b^−^), and DC‐D (CD103^+^ CD11b^+^) was compared between total DCs and STM^+^ DCs (B). Error bars indicate mean ± *SEM*. Statistical significance was analyzed using two‐way ANOVA with Bonferroni's posttest. As mentioned in this figure, samples with fewer than 20 STM^+^ DCs were excluded from all analyses; data shown include WT *n* = 5, CCL17^E/+^ *n* = 12, CCL17^E/E^ *n* = 6, pooled from five independent experiments

Intestinal DCs can be subdivided into four different subpopulations based on the expression of CD103 and CD11b according to Cerovic et al.^37^ In previous studies all DC subsets have been shown to carry STM within the mLN.^14^, ^33^ In line, we also observed that all DC subtypes contained STM, but the CD103^+^ CD11b^−^ DC subset (DC‐C) was disproportionally enriched among the STM‐carrying cells (Figure 3A). Approximately 56% of all STM^+^ DC had the CD103^+^ CD11b^−^ phenotype, which was a modest but significant enrichment compared with the normal representation of this DC subset, where CD103^+^ CD11b^−^ DC (DC‐C) represented approximately 42% of total DCs (Figure 3B; *p* < .001). The higher frequency of STM residing in CD103^+^ CD11b^−^ DCs (DC‐C) occurred independently of CCL17 expression (Figure 3B). These findings suggest that all DC subsets harbor STM, with an overrepresentation of the CD103^+^ CD11b^−^ DC (DC‐C) subset. Finally, the STM distribution was largely unaffected by the presence or absence of CCL17.

### 3.4 CCL17 expression increases in specific intestinal DC subsets after *Salmonella* Typhimurium infection

**Figure 4:**
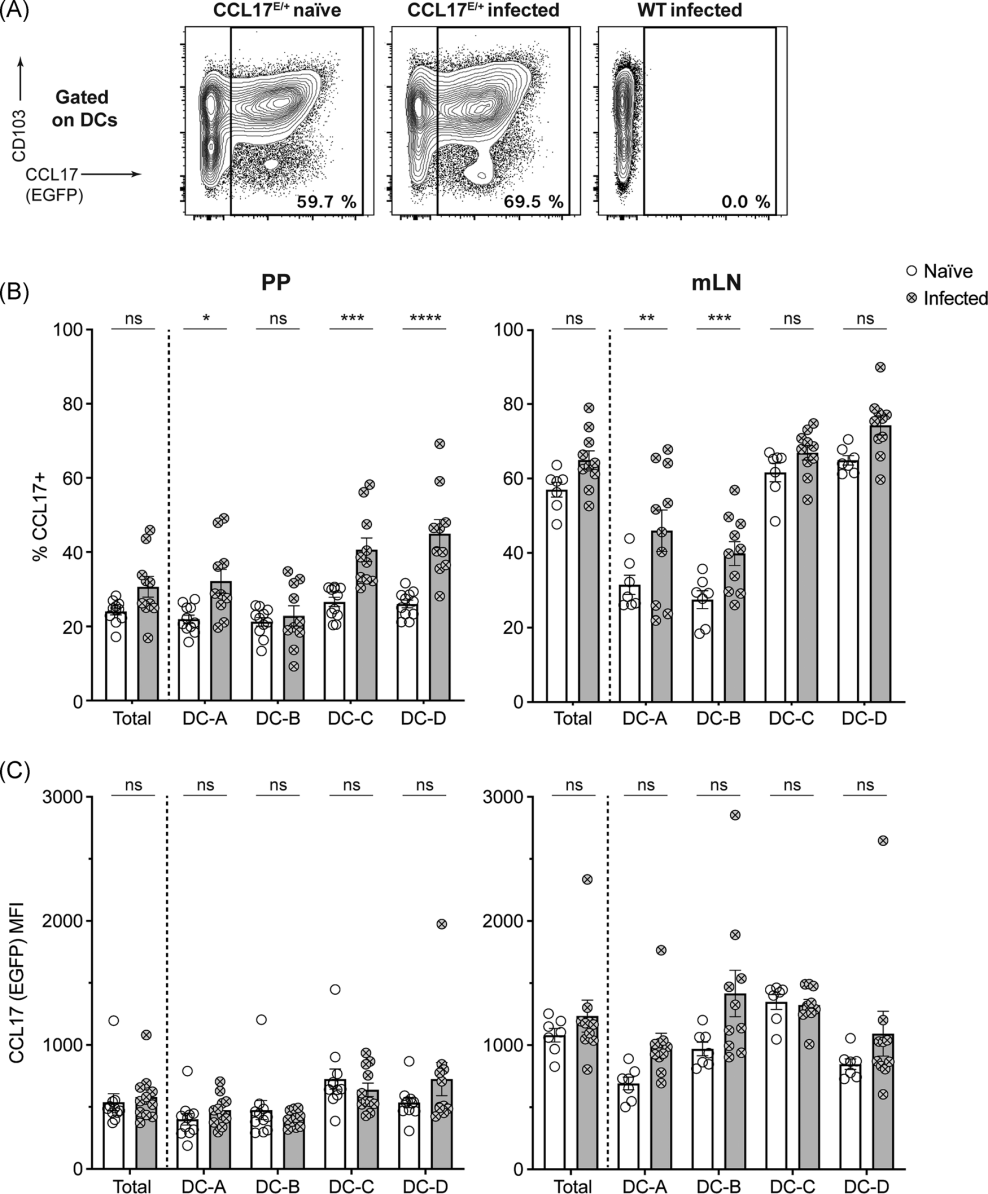
CCL17 expression increases in specific intestinal DC subsets after *Salmonella* Typhimurium infection. CCL17^E/+^ mice were infected via oral gavage with 1 × 10^9^ STM. PP and mLN were collected, single‐cell suspensions were stained for DC surface markers, and subjected to flow cytometry 19 h postinfection. DC was identified as MHCII^+^ CD64^−^ CD11c^+^ cells and was further subdivided into four subpopulations depending on their expression of CD103 and CD11b. Representative FACS plots show the frequency (%) of CCL17^+^ DC (A). Bar graphs show CCL17^+^ cell frequencies of total DCs and DC subsets (B) and mean fluorescence intensity (MFI) of CCL17 expression in CCL17^+^ total DCs and DC subsets (C). Statistical significance was tested using one‐way ANOVA with Tukey's posttest for multiple comparisons. Error bars indicate mean ± *SEM* (*n* = 11, pooled from three independent experiments)

The analysis showed that CCL17‐expressing DCs are located at the site of STM uptake in the PP and are, at least in part, infected with bacteria, hence the level of CCL17 expression was examined to test whether the production of the chemokine is affected by STM infection. CCL17^E/+^ reporter mice were orally gavaged with STM SL1344. PP and mLN were isolated 19 h postinfection and expression levels of CCL17/EGFP in intestinal DCs were analyzed via flow cytometry. FACS analysis revealed a significant increase in the proportion of specific DC subsets that expressed CCL17 in the mLN and in the PP after STM infection (Figure 4). In PP, the DC‐A subset, and especially the DC‐C and DC‐D subpopulations showed a significant increase in the frequency of CCL17‐expressing cells compared with naïve controls (Figure 4A,B). In mLN, there were similar increases in the frequency of CCL17 expression in DC‐A and DC‐B but not in DC‐C and DC‐D, indicating that infection with STM resulted in an increased frequency of CCL17^+^ cells in some DC subsets. As depicted in Figure 4C, the level of CCL17 expression per cell as determined by the mean fluorescence intensity (MFI) was not significantly altered.

Interestingly, the DC subsets that expressed CD103 (DC‐C and DC‐D) were the subsets that showed the strongest expansion of CCL17^+^ cells after STM challenge in PP. In contrast, in the mLN, the CD103^−^ DC subsets demonstrated an increase in CCL17^+^ cells. These observations suggested a differential STM‐induced expression of CCL17 in different DC subsets, depending on the organ analyzed. It should be noted though that the CD103^+^ DC subsets of mLN constitutively express high levels of CCL17 under homeostatic conditions.

### 3.5 CCL17 does not substantially contribute to the dissemination of *Salmonella* Typhimurium

**Figure 5:**
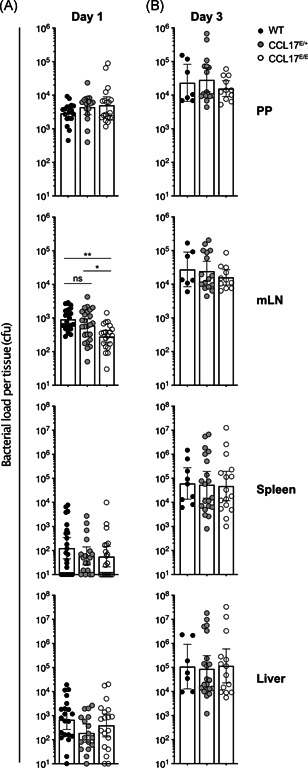
Bacterial load in PP, mLN, spleen, and liver depending on *Ccl17* genotype. Wt, CCL17^E/+^, and CCL17^E/E^ mice were infected via oral gavage with 1 × 10^9^ STM. Graphs show the numbers of Salmonella colony forming units (CFUs) recovered from homogenized PP, mLN, spleen, and liver 1 (A) and 3 (B) days postinfection. Statistical significance was tested using one‐way ANOVA with Tukey's posttest for multiple comparisons. Error bars indicate geometric mean with 95% CI (Day 1: *n* = 16–23, pooled from seven independent experiments; Day 3: *n* = 7–20, pooled from five to seven independent experiments)

To examine whether the absence of CCL17 affected the spread of STM from the gut to the mLN and then to systemic organs, Wt, CCL17^E/+^ reporter, and CCL17‐deficient CCL17^E/E^ mice were orally gavaged with STM SL1344. The PP, mLN, spleen, and liver were isolated 1 or 3 days postinfection and evaluated for bacterial counts. STM was detected on Day 1 postinfection in all organs analyzed, with PP having the highest counts, consistent with oral administration of the bacteria (Figure 5A). CFU counts from Day 1 did not vary significantly between mouse strains in the PP, spleen or liver, but were reduced in the mLN of CCL17‐deficient mice compared with wt and CCL17^E/+^ controls (Figure 5A), where there was a fourfold increase in the wt mice compared with the CCL17‐deficient CCL17^E/E^ mice. No significant differences could be detected between wt and CCL17^E/+^ controls (Figure 5A). In all four organs, CFU counts from Day 3 were increased compared to Day 1, but no differences were detected between mouse strains in mLN or the other organs that were investigated (Figure 5B).

Altogether, these results demonstrate a statistically significant, yet modest influence of CCL17 on the spreading of STM from the PP to mLN, but not to systemic organs, in the early phase of infection after oral administration of the bacteria.

## 4 DISCUSSION

In this study, we investigated the influence of the pro‐inflammatory chemokine CCL17 on the uptake and dissemination of STM during the early critical phase of infection when bacteria leave the GI tract to initiate systemic infection. We showed that CCL17‐expressing cells localize in the vicinity of STM in the SED of PP and that bacterial infection triggered upregulation of CCL17 expression in specific intestinal DC subsets in a tissue‐specific manner. Further, our work reveals that the proportion of CCL17^+^ DCs was significantly higher in STM^+^ cells compared with noninfected cells. Nevertheless, deficiency in CCL17 resulted only in a moderate reduction of the bacterial burden in the mLN but not in PP or systemic organs one day after STM infection. These findings indicate that CCL17‐production in MNP is not essential for early dissemination of STM from the intestine to systemic organs.

In previous studies of naïve CCL17^E/+^ reporter mice, we documented the presence of CCL17‐expressing cells in the SED of PP as well as in the T cell zone of PP and mLN.^22^ DCs have been identified as the main source of CCL17 in several organs, including the mLN.^22^, ^38^ Hence, the observation that CCL17^+^ cells are found in the SED of PP adjacent to STM supported the hypothesis that these cells take up bacteria that have breached the epithelial barrier. The PP is the primary site of intestinal invasion by *Salmonella* 1, 31 and both Mφ and DC located in the SED have been described to take up STM and therefore have been ascribed the role of “Trojan Horse” for STM.^10^, ^32^

From the intestine, some of the DCs that migrate to draining LN carry STM,^12^, ^14^, ^33^ shielding the bacteria from the immune system. We found that CCL17^+^ DCs colocated with STM within mLN. Bacteria, however, were not restricted to CCL17^+^ DCs. Previous studies demonstrating DCs as the cell type responsible for the transport of STM from the gut to the mLN,^14^, ^33^ described the CD103^+^ CD11b^+^ DC subset as the main population harboring STM in the mLN. In contrast, data presented here suggested that the CD103^+^ CD11b^−^ DC subset was overrepresented in STM^+^ DCs compared to all DCs in the mLN. The differences between the present study and previous studies most likely result from the different STM‐infection models used. Bogunovic et al.^33^ and Bravo‐Blas et al.^14^ used mice pretreated with streptomycin before oral infection, in contrast to the present study where mice were directly infected with virulent wt STM without prior antibiotic treatment. In the streptomycin model, STM replicates in the GI tract following inhibition of the gut microbiome by streptomycin to very high numbers,^39^ and enter the intestinal mucosa not only through the PP but also directly via enterocytes along the intestinal lining because of increased intestinal replication in a reduced microbiome environment.^40^ In the absence of antibiotic pretreatment, however, STM enter preferentially via the PP.^1^, ^31^ Interestingly, all three studies, including the present, demonstrated that STM is not found exclusively within one single DC subpopulation, and the bacteria likely exploit multiple immune cell subsets as “carriers” for systemic dissemination.

DCs are the main producers of CCL17, which is known as a homeostatic and inflammatory chemokine.^22^, ^27^ Only limited information is available on its expression after bacterial infection. Here, in a flow cytometry analysis of different myeloid cells and with a particular focus on DC subsets, it was demonstrated that CCL17^+^ DCs not only carried STM and contributed to the transmission of bacteria but also that the expression of CCL17/EGFP was upregulated upon infection. In line with these observations, TLR‐stimulation via LPS or other PAMP (CpG‐ODN, Poly‐(I:C)), as well as TNF‐α induces CCL17 expression.^22^, ^38^, ^41^ Of note, CCL17 expression increased more strongly in the CD103^+^ DC subsets in PP, whereas in the mLN, the CD103^−^ DC subsets were more affected. As CD103 is known to be involved in the retention of tissue‐resident T cells,^42^, ^43^, ^44^ downregulation of CD103 may to some extent facilitate migration of intestinal DC from the PP to the lymph nodes. Interestingly, human monocyte‐derived DCs downregulate CD103 upon bacterial infection.^45^ In fact, besides CD103^+^ DCs, CD103^−^ DCs were also shown to migrate in the lymph.^37^, ^46^ These cells may display a stronger upregulation of CCL17 upon arrival in the mLN. It should be noted in this context that CD103/CD11b double negative DCs corresponding to the DC‐A subset are considered to be the main DC population transporting antigens from the PP to the mLN, whereas the other three subsets may preferentially migrate to the mLN from the lamina propria.^46^ As STM preferentially enters the intestinal mucosa via the PP in mice, migration of STM carrying DC from the lamina propria to the mLN is probably not the main route of bacterial transmission in our experiments.

The chemokine CCL17 is required for CCR7‐dependent DC migration in the skin.^26^ If this was also true for intestinal DC migration, CCL17 deficiency might result in decreased bacterial loads in mLN. However, we observed only a small (i.e. fourfold) reduction in bacterial load in mLN 1 day after oral infection of CCL17‐deficient mice with STM. Since STM can also be transported from the intestine to the mLN as free bacteria within the lymph,^14^ impaired DC migration may not lead to a large decrease in bacterial load in the draining LN, depending on the contribution of these extracellular bacteria to LN infection. For comparison, Voedisch et al.^12^ demonstrated an impaired DC migration in STM infected CCR7^−/−^ animals, which led to a tenfold reduction of the bacterial load in the mLN, but not in the liver or spleen of CCR7^−/−^ mice. These findings indicate that CCL17‐expressing DCs play a redundant role in the early spread of the bacteria from the intestine to the draining LN, and may only be of minor importance for bacteria to then reach systemic sites. This is probably because STM may also be transported by cell types other than DCs (e.g., monocytes), and/or STM might enter the circulation independently of DCs or other myeloid cells.

Nevertheless, DCs represent perfect “Trojan horses” in which STM can survive,^47^, ^48^ and disseminate to systemic tissues by exploiting the intrinsic migratory capacity of DCs, while mostly hidden from detection by the effector immune response. With their unique ability to prime antigen‐specific naïve T cells, DCs are vital for initiating adaptive immunity in response to infection. CCL17^+^ DCs have proven to be very efficient in T cell priming in particular inducing IFN‐γ‐secreting T cells.^22^ The essential role of helper T cells in immunity against STM is well established. Multiple studies have shown substantial expansion of STM‐specific CD4^+^ T cells and prompt gain of Th1 effector functions, characterized by the ability to secrete IFN‐γ and TNF‐α upon restimulation.^49^, ^50^ Mice deficient in Th1 cells, due to a lack of the transcription factor T‐bet, are unable to resolve primary, acute *Salmonella* infection^51^ as are CD4^+^ T cell‐deficient mice.^52^ Thus, it is possible that activated CCL17^+^ DCs not only participate in the spreading of STM from the intestine to the mLN, but also function as potent inducers of an STM‐specific Th1 T cell response. To study this hypothesis, infections with attenuated STM would need to be performed, since infection with wild‐type STM, as done in this study, leads to severe disease symptoms within a week from infection and before effective adaptive immunity would have time to develop.

## 5 CONCLUSION

In summary, the present work broadens our knowledge of the phenotype and localization of CCL17‐producing cells in the context of *Salmonella* infection. In addition, the differential upregulation of CCL17 in distinct intestinal DC subsets in response to *Salmonella* infection was revealed. Understanding the role of the various myeloid cell populations involved in the defense against the intestinal pathogen *Salmonella* Typhimurium will shed light on how these cells can be exploited for early control of the infection and, ultimately, in initiating protective immunity against intestinal bacteria.

## CONFLICT OF INTERESTS

The authors declare that there are no conflict of interests.

## AUTHOR CONTRIBUTIONS

*Conceptualization*: Anna B. Erazo, Richard A. Strugnell, and Irmgard Förster. *Methodology*: Anna B. Erazo, Nancy Wang, Lorenz Fülle, Richard A. Strugnell, and Irmgard Förster. *Experimentation and data analysis*: Anna B. Erazo, Lena Standke, Adrian D. Semeniuk, Lorenz Fülle, Sevgi C. Cengiz, Manja Thiem, and Nancy Wang. *Resources*: Heike Weighardt, Nancy Wang, Richard A. Strugnell, and Irmgard Förster. *Writing—original draft preparation*: Anna B. Erazo and Irmgard Förster. *Writing—review and editing*: Anna B. Erazo, Lorenz Fülle, Heike Weighardt, Nancy Wang, Richard A. Strugnell, and Irmgard Förster. *Data visualization*: Anna B. Erazo and Nancy Wang. *Supervision*: Nancy Wang, Richard A. Strugnell, and Irmgard Förster. *Funding acquisition*: Richard A. Strugnell and Irmgard Förster.

## Supporting information

Supplementary information.

## Data Availability

The data that support the findings of this study are available from the corresponding author upon reasonable request.

## References

[1] Carter PB , Collins FM . The route of enteric infection in normal mice. J Exp Med. 1974;139:1189‐1203. 4596512 10.1084/jem.139.5.1189 PMC2139651

[2] Gordon MA . *Salmonella* infections in immunocompromised adults. J Infect. 2008;56:413‐422. 18474400 10.1016/j.jinf.2008.03.012

[3] Feasey NA , Dougan G , Kingsley RA , Heyderman RS , Gordon MA . Invasive non‐typhoidal *Salmonella* disease: an emerging and neglected tropical disease in Africa. Lancet. 2012;379:2489‐2499. 22587967 10.1016/S0140-6736(11)61752-2 PMC3402672

[4] Gilchrist JJ , MaClennan CA , Hill AVS . Genetic susceptibility to invasive *Salmonella* disease. Nat Rev Immunol. 2015;15:452‐463. 26109132 10.1038/nri3858

[5] Okoro CK , Kingsley RA , Connor TR , et al. Intracontinental spread of human invasive *Salmonella* Typhimurium pathovariants in sub‐Saharan Africa. Nat Genet. 2012;44:1215‐1221. 23023330 10.1038/ng.2423 PMC3491877

[6] Strugnell RA , Scott TA , Wang N , et al. *Salmonella* vaccines: lessons from the mouse model or bad teaching? Curr Opin Microbiol. 2014;17:99‐105. 24440968 10.1016/j.mib.2013.12.004

[7] Broz P , Ohlson MB , Monack DM . Innate immune response to *Salmonella* Typhimurium, a model enteric pathogen. Gut Microbes. 2012;3:62‐70. 22198618 10.4161/gmic.19141 PMC3370950

[8] Gayet R , Bioley G , Rochereau N , Paul S , Corthésy B . Vaccination against *Salmonella* infection: the Mucosal Way. Microbiol Mol Biol Rev. 2017;81:1‐26. 10.1128/MMBR.00007-17 PMC5584317 28615285

[9] Palmer AD , Slauch JM . Mechanisms of *Salmonella* pathogenesis in animal models. Hum Ecol Risk Assess. 2017;23:1877‐1892. 31031557 10.1080/10807039.2017.1353903 PMC6484827

[10] Niess JH , Brand S , Gu X , et al. CX3CR1‐mediated dendritic cell access to the intestinal lumen and bacterial clearance. Science. 2005;307:254‐258. 15653504 10.1126/science.1102901

[11] Galan JE , Curtiss R . Cloning and molecular characterization of genes whose products allow *Salmonella* Typhimurium to penetrate tissue culture cells. Proc Natl Acad Sci USA. 1989;86:6383‐6387. 2548211 10.1073/pnas.86.16.6383 PMC297844

[12] Voedisch S , Koenecke C , David S , et al. Mesenteric lymph nodes confine dendritic cell‐mediated dissemination of *Salmonella enterica* serovar Typhimurium and limit systemic disease in mice. Infect Immun. 2009;77:3170‐3180. 19506012 10.1128/IAI.00272-09 PMC2715677

[13] Bogunovic M , Ginhoux F , Helft J , et al. Origin of the lamina propria dendritic cell network. Immunity. 2009;31:513‐525. 19733489 10.1016/j.immuni.2009.08.010 PMC2778256

[14] Bravo‐Blas A , Utriainen L , Clay SL , et al. *Salmonella enterica* serovar Typhimurium travels to mesenteric lymph nodes both with host cells and autonomously. J Immunol. 2019;202:260‐267. 30487173 10.4049/jimmunol.1701254 PMC6305795

[15] Burton NA , Schürmann N , Casse O , et al. Disparate impact of oxidative host defenses determines the fate of salmonella during systemic infection in mice. Cell Host Microbe. 2014;15:72‐83. 24439899 10.1016/j.chom.2013.12.006

[16] Loetscher Y , Wieser A , Lengefeld J , et al. Salmonella transiently reside in luminal neutrophils in the inflamed gut. PLOS One. 2012;7. 10.1371/journal.pone.0034812 PMC3321032 22493718

[17] Monack DM , Bouley DM , Falkow S . *Salmonella typhimurium* persists within macrophages in the mesenteric lymph nodes of chronically infected Nramp1^+/+^ mice and can be reactivated by IFNγ neutralization. J Exp Med. 2004;199:231‐241. 14734525 10.1084/jem.20031319 PMC2211772

[18] Richter‐Dahlfors A , Buchan AMJ , Finlay BB . Murine salmonellosis studied by confocal microscopy: *Salmonella typhimurium* resides intracellularly inside macrophages and exerts a cytotoxic effect on phagocytes in vivo. J Exp Med. 1997;186:569‐580. 9254655 10.1084/jem.186.4.569 PMC2199036

[19] Mastroeni P , Grant A , Restif O , Maskell D . A dynamic view of the spread and intracellular distribution of *Salmonella enterica* . Nat Rev Microbiol. 2009;7:73‐80. 19079353 10.1038/nrmicro2034

[20] Bayer‐Santos E , Durkin CH , Rigano LA , et al. The *Salmonella* effector SteD mediates MARCH8‐dependent ubiquitination of MHC II molecules and inhibits T Cell Activation. Cell Host Microbe. 2016;20:584‐595. 27832589 10.1016/j.chom.2016.10.007 PMC5104694

[21] Imai T , Yoshida T , Baba M , Nishimura M , Kakizaki M , Yoshie O . Molecular cloning of a novel T cell‐directed CC chemokine expressed in thymus by signal sequence trap using Epstein‐Barr virus vector. J Biol Chem. 1996;271:21514‐21521. 8702936 10.1074/jbc.271.35.21514

[22] Alferink J , Lieberam I , Reindl W , et al. Compartmentalized production of CCL17 in vivo: strong inducibility in peripheral dendritic cells contrasts selective absence from the spleen. J Exp Med. 2003;197:585‐599. 12615900 10.1084/jem.20021859 PMC2193819

[23] Fülle L , Steiner N , Funke M , et al. RNA aptamers recognizing murine CCL17 inhibit T cell chemotaxis and reduce contact hypersensitivity in vivo. Mol Ther. 2018;26:95‐104. 29103909 10.1016/j.ymthe.2017.10.005 PMC5763148

[24] Weber C , Meiler S , Döring Y , et al. CCL17‐expressing dendritic cells drive atherosclerosis by restraining regulatory T cell homeostasis in mice. J Clin Invest. 2011;121:2898‐2910. 21633167 10.1172/JCI44925 PMC3223829

[25] Semmling V , Lukacs‐Kornek V , Thaiss CA , et al. Alternative cross‐priming through CCL17‐CCR4‐mediated attraction of CTLs toward NKT cell‐licensed DCs. Nat Immunol. 2010;11:313‐320. 20190758 10.1038/ni.1848

[26] Stutte S , Quast T , Gerbitzki N , et al. Requirement of CCL17 for CCR7‐ and CXCR4‐dependent migration of cutaneous dendritic cells. Proc Natl Acad Sci USA. 2010;107:8736‐8741. 20421491 10.1073/pnas.0906126107 PMC2889308

[27] Zlotnik A , Yoshie O . The chemokine superfamily revisited. Immunity. 2012;36:705‐712. 22633458 10.1016/j.immuni.2012.05.008 PMC3396424

[28] Achuthan A , Cook AD , Lee M‐C , et al. Granulocyte macrophage colony‐stimulating factor induces CCL17 production via IRF4 to mediate inflammation. J Clin Investig. 2016:126. 10.1172/JCI87828 PMC5004969 27525438

[29] Heiseke AF , Faul AC , Lehr HA , et al. CCL17 promotes intestinal inflammation in mice and counteracts regulatory T cell‐mediated protection from colitis. Gastroenterology. 2012;142:335‐345. 22057112 10.1053/j.gastro.2011.10.027

[30] Knodler LA , Crowley SM , Sham HP , et al. Noncanonical inflammasome activation of caspase‐4/caspase‐11 mediates epithelial defenses against enteric bacterial pathogens. Cell Host Microbe. 2014;16:249‐256. 25121752 10.1016/j.chom.2014.07.002 PMC4157630

[31] Jones BD , Ghori N , Falkow S . *Salmonella typhimurium* initiates murine infection by penetrating and destroying the specialized epithelial M cells of the peyer′s patches. J Exp Med. 1994;180:15‐23. 8006579 10.1084/jem.180.1.15 PMC2191576

[32] Hopkins SA , Niedergang F , Corthesy‐Theulaz IE , Kraehenbuhl JP . A recombinant *Salmonella typhimurium* vaccine strain is taken up and survives within murine Peyer′s patch dendritic cells. Cell Microbiol. 2000;2:59‐68. 11207563 10.1046/j.1462-5822.2000.00035.x

[33] Bogunovic M , Ginhoux F , Helft J , et al. Origin of the lamina propria dendritic cell network. Immunity. 2009;18:513‐525. 10.1016/j.immuni.2009.08.010 PMC2778256 19733489

[34] Vazquez‐Torres A , Jones‐Carson J , Bäumler AJ , et al. Extraintestinal dissemination of *Salmonella* by CD18‐expressing phagocytes. Nature. 1999;401:804‐808. 10548107 10.1038/44593

[35] Houston SA , Cerovic V , Thomson C , Brewer J , Mowat AM , Milling S . The lymph nodes draining the small intestine and colon are anatomically separate and immunologically distinct. Mucosal Immunol. 2016;9:468‐478. 26329428 10.1038/mi.2015.77

[36] Bosteels C , Neyt K , Vanheerswynghels M , et al. Inflammatory type 2 cDCs acquire features of cDC1s and macrophages to orchestrate immunity to respiratory virus infection. Immunity. 2020;52:1039‐1056.e9. 32392463 10.1016/j.immuni.2020.04.005 PMC7207120

[37] Cerovic V , Houston SA , Scott CL , et al. Intestinal CD103^−^ dendritic cells migrate in lymph and prime effector T cells. Mucosal Immunol. 2013;6:104‐113. 22718260 10.1038/mi.2012.53

[38] Globisch T , Steiner N , Fülle L , et al. Cytokine‐dependent regulation of dendritic cell differentiation in the splenic microenvironment. Eur J Immunol. 2014;44:500‐510. 24136200 10.1002/eji.201343820

[39] Kaiser P , Diard M , Stecher B , Hardt W‐D . The streptomycin mouse model for *Salmonella* diarrhea: functional analysis of the microbiota, the pathogen′s virulence factors, and the host′s mucosal immune response. Immunol Rev. 2012;245:56‐83. 22168414 10.1111/j.1600-065X.2011.01070.x

[40] Barthel M , Hapfelmeier S , Quintanilla‐Martínez L , et al. Pretreatment of mice with streptomycin provides a *Salmonella enterica* serovar Typhimurium colitis model that allows analysis of both pathogen and host. Infect Immun. 2003;71:2839‐2858. 12704158 10.1128/IAI.71.5.2839-2858.2003 PMC153285

[41] Fülle L , Offermann N , Hansen JN , et al. CCL17 exerts a neuroimmune modulatory function and is expressed in hippocampal neurons. GLIA. 2018;66:2246‐2261. 30277599 10.1002/glia.23507

[42] Sheridan BS , Lefrançois L . Regional and mucosal memory T cells. Nat Immunol. 2011;12:485‐491. 21739671 10.1038/ni.2029 PMC3224372

[43] Wakim LM , Woodward‐Davis A , Bevan MJ . Memory T cells persisting within the brain after local infection show functional adaptations to their tissue of residence. Proc Natl Acad Sci USA. 2010;107:17872‐17879. 20923878 10.1073/pnas.1010201107 PMC2964240

[44] Mackay LK , Stock AT , Ma JZ , et al. Long‐lived epithelial immunity by tissue‐resident memory T (TRM) cells in the absence of persisting local antigen presentation. Proc Natl Acad Sci USA. 2012;109:7037‐7042. 22509047 10.1073/pnas.1202288109 PMC3344960

[45] Roe MM , Swain S , Sebrell TA , et al. Differential regulation of CD103 (αE integrin) expression in human dendritic cells by retinoic acid and Toll‐like receptor ligands. J Leukoc Biol. 2017;101:1169‐1180. 28087652 10.1189/jlb.1MA0316-131R PMC5380378

[46] Worbs T , Hammerschmidt SI , Förster R . Dendritic cell migration in health and disease. Nat Rev Immunol. 2017;17:30‐48. 27890914 10.1038/nri.2016.116

[47] Bueno SM , González PA , Carreño LJ , et al. The capacity of *Salmonella* to survive inside dendritic cells and prevent antigen presentation to T cells is host specific. Immunology. 2008;124:522‐533. 18266715 10.1111/j.1365-2567.2008.02805.x PMC2492944

[48] Niedergang F , Sirard JC , Blanc CT , Kraehenbuhl JP . Entry and survival of *Salmonella typhimurium* in dendritic cells and presentation of recombinant antigens do not require macrophage‐specific virulence factors. Proc Natl Acad Sci USA. 2000;97:14650‐14655. 11121065 10.1073/pnas.97.26.14650 PMC18973

[49] Mittrücker HW , Köhler A , Kaufmann SHE . Characterization of the murine T‐lymphocyte response to *Salmonella enterica* serovar Typhimurium infection. Infect Immun. 2002;70:199‐203. 11748183 10.1128/IAI.70.1.199-203.2002 PMC127597

[50] Srinivasan A , Foley J , McSorley SJ . Massive number of antigen‐specific CD4 T cells during vaccination with live attenuated salmonella causes interclonal competition. J Immunol. 2004;172:6884‐6893. 15153507 10.4049/jimmunol.172.11.6884

[51] Ravindran R , Foley J , Stoklasek T , Glimcher LH , McSorley SJ . Expression of T‐bet by CD4 T cells is essential for resistance to *Salmonella* infection. J Immunol. 2005;175:4603‐4610. 16177105 10.4049/jimmunol.175.7.4603

[52] Kupz A , Bedoui S , Strugnell RA . Cellular requirements for systemic control of *Salmonella enterica* serovar Typhimurium infections in mice. Infect Immun. 2014;82:4997‐5004. 25225248 10.1128/IAI.02192-14 PMC4249260

