# Supplementary material for: CCL17‐expressing dendritic cells in the intestine are preferentially infected by Salmonella but CCL17 plays a redundant role in systemic dissemination
Source: Immun Inflamm Dis. 2021 May 4;9(3):891–904. doi: 10.1002/iid3.445 (PMC8342217; doi:10.1002/iid3.445)
Supplement: Supplementary file 1 — Supplementary information. [file IID3-9-891-s001.pdf]

Supplementary data

CCL17-expressing dendritic cells in the intestine are preferentially infected by *Salmonella* but CCL17 plays a redundant role in systemic dissemination

Running title: CCL17 in *Salmonella* infection

Anna Belen Erazo[1,2,#], Nancy Wang[2,#], Lena Standke[3], Adrian David Semeniuk[1,2], Lorenz Fülle[1], Sevgi Can Cengiz[1], Manja Thiem[1], Heike Weighardt[1], Richard Anthony Strugnell[2*], Irmgard Förster[1*]

[1] Immunology and Environment, Life and Medical Sciences (LIMES) Institute, University of Bonn, Bonn, Germany

[2] The Department of Microbiology and Immunology, The University of Melbourne at the Peter Doherty Institute for Infection and Immunity, Melbourne, Victoria, Australia

[3] Institute of Innate Immunity, University Hospital Bonn, Medical Faculty, Bonn, Germany

[#] A.B.E. and N.W. should be considered joint first author
*R.A.S. and I.F. should be considered joint senior author

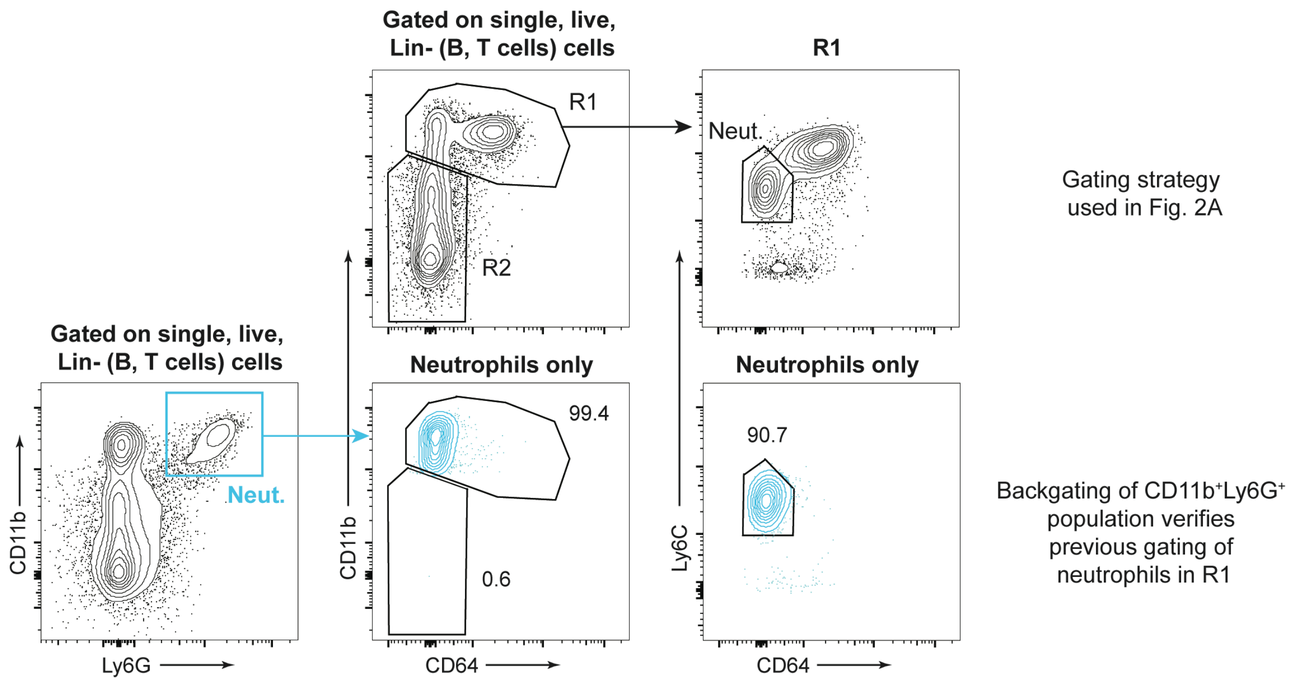

Supplementary Figure 1. *Flow cytometry analysis verifies exclusion of neutrophils in analysis of other myeloid cell populations.* Wt mice were infected with 1×10^9 cfu STM via oral gavage. Spleen and mLN were isolated 36h after infection. Representative FACS plot of neutrophils ($CD11b^+Ly6G^+$, shown in blue against other cells shown in black) backgated using the same strategy shown in Fig 2A, indicating that the vast majority of neutrophils aggregated in the R1 gate and can be further characterised as $Ly6C^{int}CD64^{neg}$.

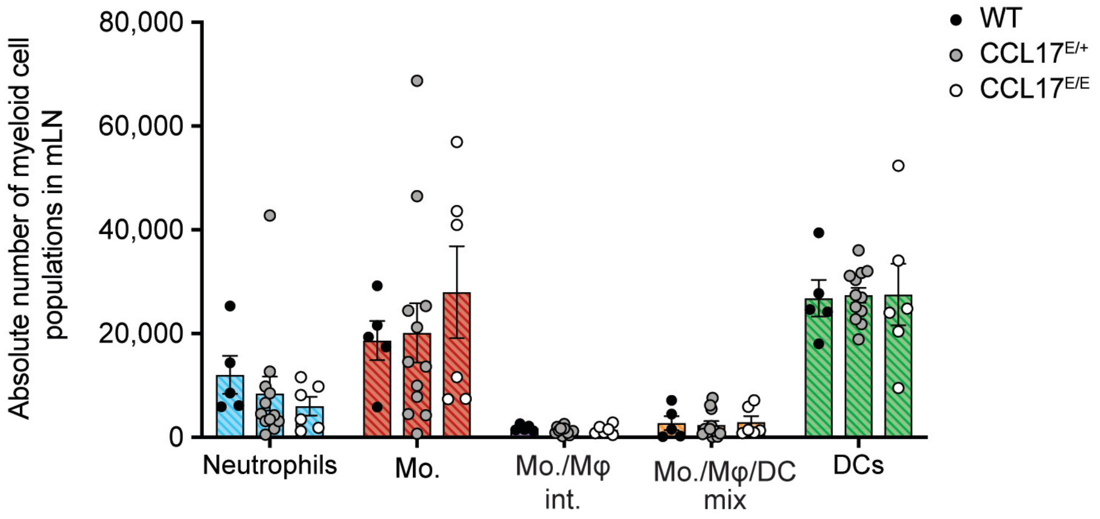

Supplementary Figure 2. *DCs and monocytes are among the most abundant myeloid cell populations after Salmonella Typhimurium infection*. Wt, CCL17$^{E/+}$, and CCL17$^{E/E}$ mice were infected with 1×10^9 STM via oral gavage. mLN were isolated from 36h-infected mice, and various myeloid cell populations were analysed and quantified using the same gating strategy as described in Fig. 2A. Shown are the absolute cell numbers of each population in mLN per mouse. Error bars indicate mean±SEM. Statistical significance was testing using two-way ANOVA with Bonferroni's post-test, however there was no incidence where the multiple comparison-adjusted *p*-value was below 0.05. Consistent with Fig 2 and Fig 3, samples with fewer than 20 STM+ DCs were excluded from all analysis; data shown include WT n=5, CCL17$^{E/+}$ n=12, CCL17$^{E/E}$ n=6, pooled from five independent experiments.
